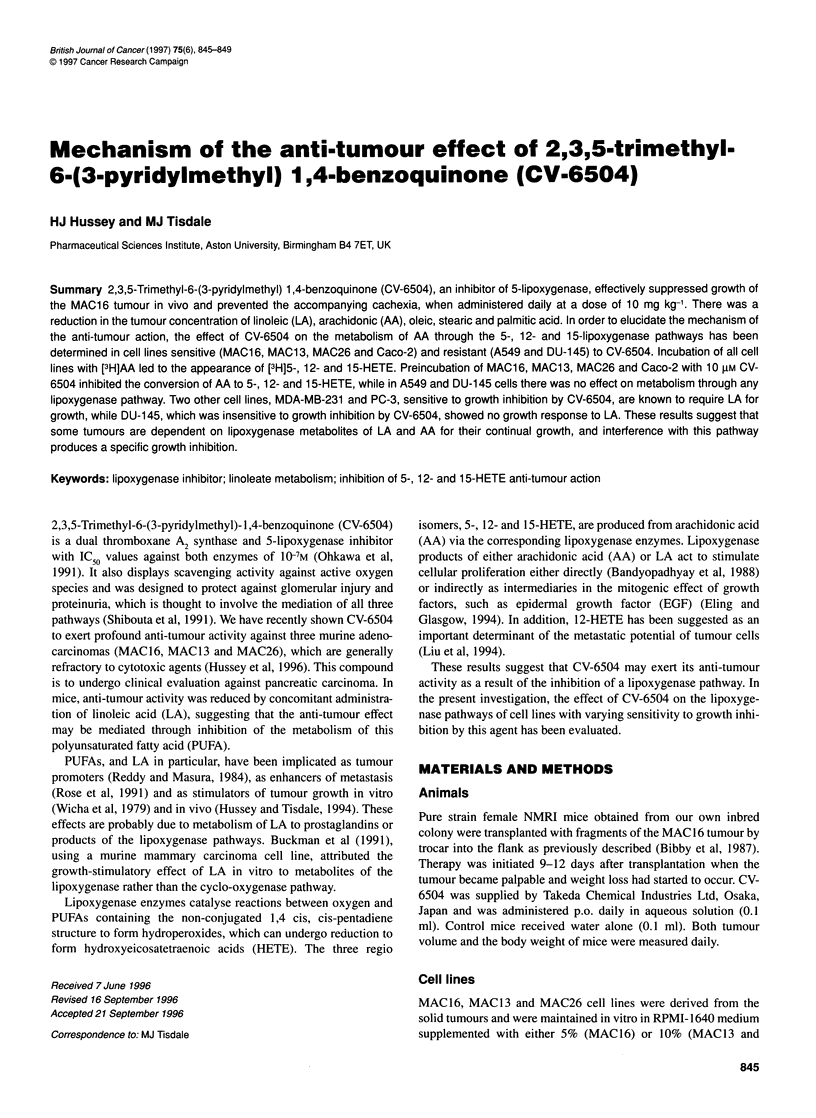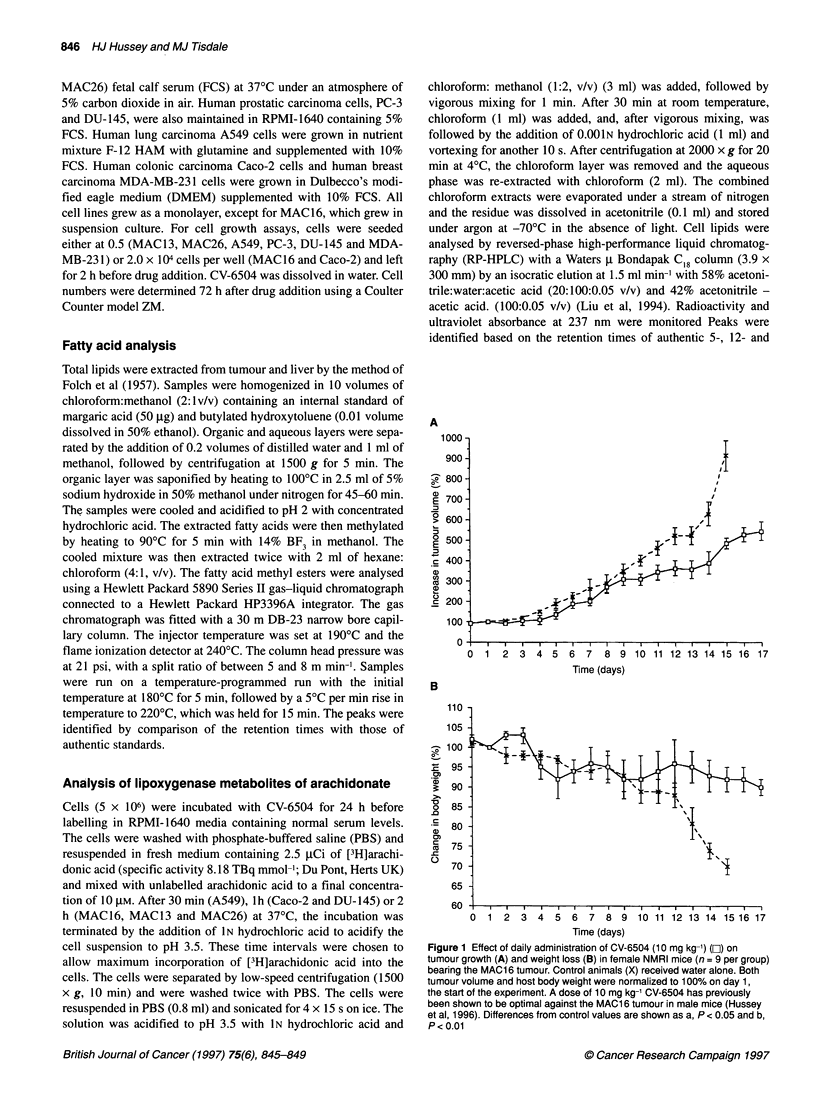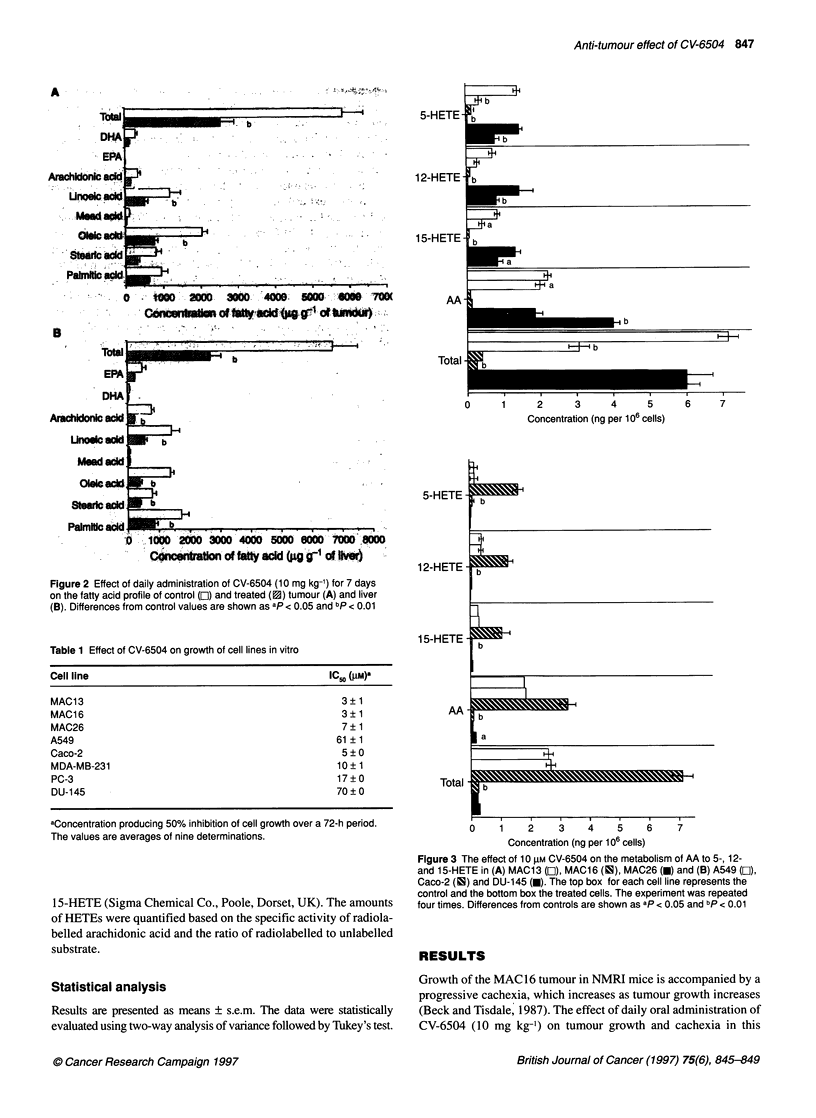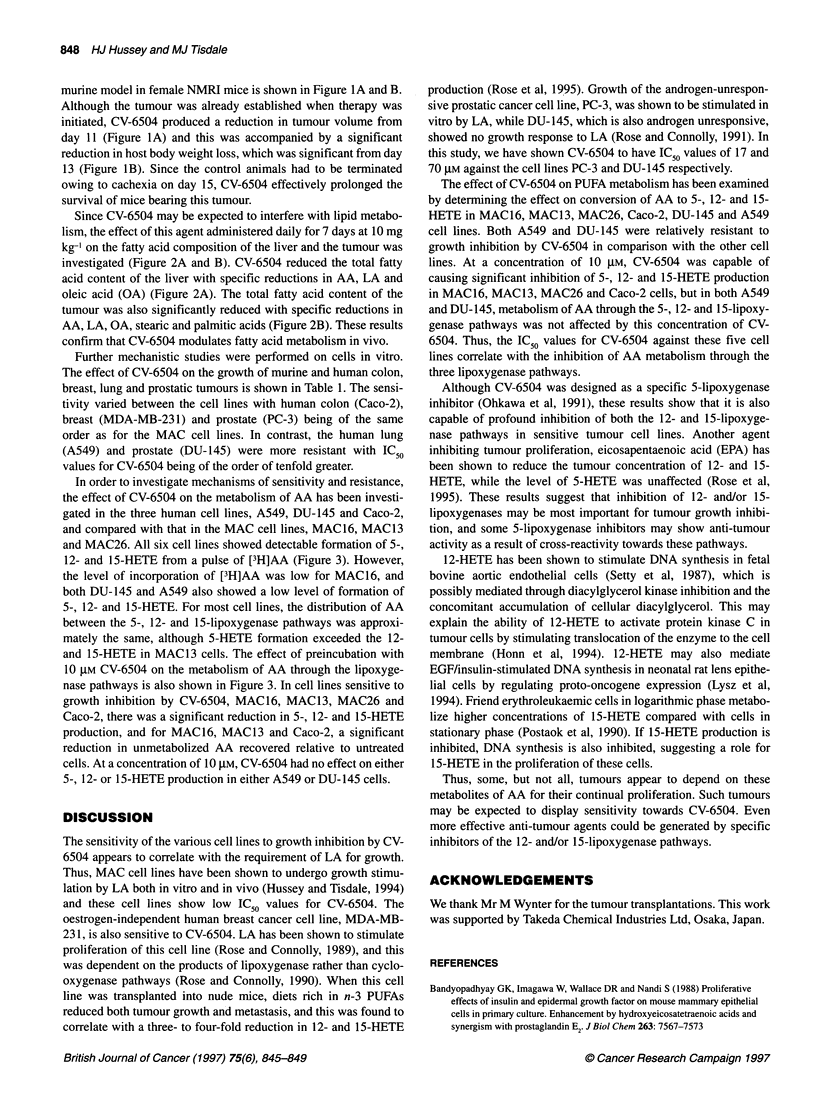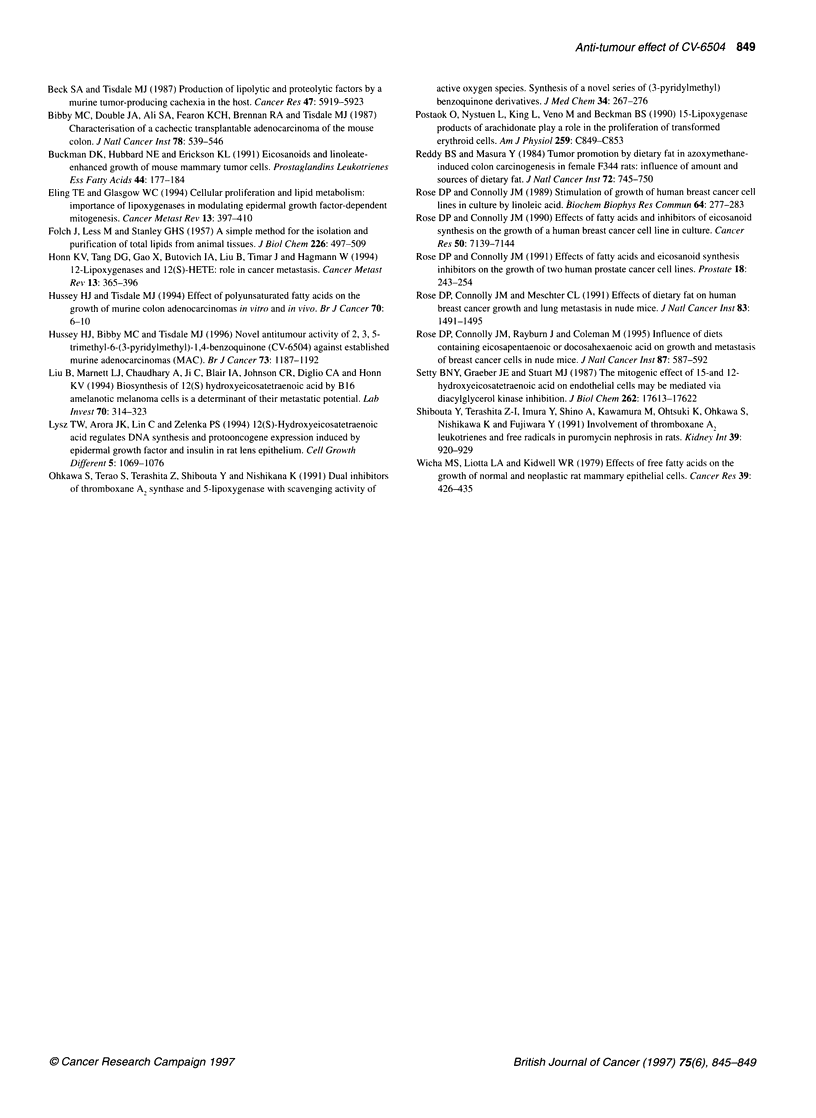# Mechanism of the anti-tumour effect of 2,3,5-trimethyl-6-(3-pyridylmethyl) 1,4-benzoquinone (CV-6504).

**DOI:** 10.1038/bjc.1997.150

**Published:** 1997

**Authors:** H. J. Hussey, M. J. Tisdale

**Affiliations:** Pharmaceutical Sciences Institute, Aston University, Birmingham, UK.

## Abstract

2,3,5-Trimethyl-6-(3-pyridylmethyl) 1,4-benzoquinone (CV-6504), an inhibitor of 5-lipoxygenase, effectively suppressed growth of the MAC16 tumour in vivo and prevented the accompanying cachexia, when administered daily at a dose of 10 mg kg(-1). There was a reduction in the tumour concentration of linoleic (LA), arachidonic (AA), oleic, stearic and palmitic acid. In order to elucidate the mechanism of the anti-tumour action, the effect of CV-6504 on the metabolism of AA through the 5-, 12- and 15-lipoxygenase pathways has been determined in cell lines sensitive (MAC16, MAC13, MAC26 and Caco-2) and resistant (A549 and DU-145) to CV-6504. Incubation of all cell lines with [3H]AA led to the appearance of [3H]5-, 12- and 15-HETE. Preincubation of MAC16, MAC13, MAC26 and Caco-2 with 10 microM CV-6504 inhibited the conversion of AA to 5-, 12- and 15-HETE, while in A549 and DU-145 cells there was no effect on metabolism through any lipoxygenase pathway. Two other cell lines, MDA-MB-231 and PC-3, sensitive to growth inhibition by CV-6504, are known to require LA for growth, while DU-145, which was insensitive to growth inhibition by CV-6504, showed no growth response to LA. These results suggest that some tumours are dependent on lipoxygenase metabolites of LA and AA for their continual growth, and interference with this pathway produces a specific growth inhibition.


British Journal of Cancer (1997) 75(6), 845–849
© 1997 Cancer Research Campaign

### Mechanism of the anti-tumour effect of 2,3,5-trimethyl-6-(3-pyridylmethyl) 1,4-benzoquinone (CV-6504)

**HJ Hussey and MJ Tisdale**

Pharmaceutical Sciences Institute, Aston University, Birmingham B4 7ET, UK

**Summary** 2,3,5-Trimethyl-6-(3-pyridylmethyl) 1,4-benzoquinone (CV-6504), an inhibitor of 5-lipoxygenase, effectively suppressed growth of the MAC16 tumour in vivo and prevented the accompanying cachexia, when administered daily at a dose of 10 mg $kg^{-1}$. There was a reduction in the tumour concentration of linoleic (LA), arachidonic (AA), oleic, stearic and palmitic acid. In order to elucidate the mechanism of the anti-tumour action, the effect of CV-6504 on the metabolism of AA through the 5-, 12- and 15-lipoxygenase pathways has been determined in cell lines sensitive (MAC16, MAC13, MAC26 and Caco-2) and resistant (A549 and DU-145) to CV-6504. Incubation of all cell lines with [$^3$H]AA led to the appearance of [$^3$H]5-, 12- and 15-HETE. Preincubation of MAC16, MAC13, MAC26 and Caco-2 with 10 μM CV-6504 inhibited the conversion of AA to 5-, 12- and 15-HETE, while in A549 and DU-145 cells there was no effect on metabolism through any lipoxygenase pathway. Two other cell lines, MDA-MB-231 and PC-3, sensitive to growth inhibition by CV-6504, are known to require LA for growth, while DU-145, which was insensitive to growth inhibition by CV-6504, showed no growth response to LA. These results suggest that some tumours are dependent on lipoxygenase metabolites of LA and AA for their continual growth, and interference with this pathway produces a specific growth inhibition.

**Keywords:** lipoxygenase inhibitor; linoleate metabolism; inhibition of 5-, 12- and 15-HETE anti-tumour action

2,3,5-Trimethyl-6-(3-pyridylmethyl)-1,4-benzoquinone (CV-6504) is a dual thromboxane $A_2$ synthase and 5-lipoxygenase inhibitor with $IC_{50}$ values against both enzymes of $10^{-7}$M (Ohkawa et al, 1991). It also displays scavenging activity against active oxygen species and was designed to protect against glomerular injury and proteinuria, which is thought to involve the mediation of all three pathways (Shibouta et al, 1991). We have recently shown CV-6504 to exert profound anti-tumour activity against three murine adenocarcinomas (MAC16, MAC13 and MAC26), which are generally refractory to cytotoxic agents (Hussey et al, 1996). This compound is to undergo clinical evaluation against pancreatic carcinoma. In mice, anti-tumour activity was reduced by concomitant administration of linoleic acid (LA), suggesting that the anti-tumour effect may be mediated through inhibition of the metabolism of this polyunsaturated fatty acid (PUFA).

PUFAs, and LA in particular, have been implicated as tumour promoters (Reddy and Masura, 1984), as enhancers of metastasis (Rose et al, 1991) and as stimulators of tumour growth in vitro (Wicha et al, 1979) and in vivo (Hussey and Tisdale, 1994). These effects are probably due to metabolism of LA to prostaglandins or products of the lipoxygenase pathways. Buckman et al (1991), using a murine mammary carcinoma cell line, attributed the growth-stimulatory effect of LA in vitro to metabolites of the lipoxygenase rather than the cyclo-oxygenase pathway.

Lipoxygenase enzymes catalyse reactions between oxygen and PUFAs containing the non-conjugated 1,4 cis, cis-pentadiene structure to form hydroperoxides, which can undergo reduction to form hydroxyeicosatetraenoic acids (HETE). The three regio isomers, 5-, 12- and 15-HETE, are produced from arachidonic acid (AA) via the corresponding lipoxygenase enzymes. Lipoxygenase products of either arachidonic acid (AA) or LA act to stimulate cellular proliferation either directly (Bandyopadhyay et al, 1988) or indirectly as intermediaries in the mitogenic effect of growth factors, such as epidermal growth factor (EGF) (Eling and Glasgow, 1994). In addition, 12-HETE has been suggested as an important determinant of the metastatic potential of tumour cells (Liu et al, 1994).

These results suggest that CV-6504 may exert its anti-tumour activity as a result of the inhibition of a lipoxygenase pathway. In the present investigation, the effect of CV-6504 on the lipoxygenase pathways of cell lines with varying sensitivity to growth inhibition by this agent has been evaluated.

### MATERIALS AND METHODS

### Animals

Pure strain female NMRI mice obtained from our own inbred colony were transplanted with fragments of the MAC16 tumour by trocar into the flank as previously described (Bibby et al, 1987). Therapy was initiated 9–12 days after transplantation when the tumour became palpable and weight loss had started to occur. CV-6504 was supplied by Takeda Chemical Industries Ltd, Osaka, Japan and was administered p.o. daily in aqueous solution (0.1 ml). Control mice received water alone (0.1 ml). Both tumour volume and the body weight of mice were measured daily.

### Cell lines

MAC16, MAC13 and MAC26 cell lines were derived from the solid tumours and were maintained in vitro in RPMI-1640 medium supplemented with either 5% (MAC16) or 10% (MAC13 and

*Received 7 June 1996*
*Revised 16 September 1996*
*Accepted 21 September 1996*

*Correspondence to:* MJ Tisdale

MAC26) fetal calf serum (FCS) at 37°C under an atmosphere of 5% carbon dioxide in air. Human prostatic carcinoma cells, PC-3 and DU-145, were also maintained in RPMI-1640 containing 5% FCS. Human lung carcinoma A549 cells were grown in nutrient mixture F-12 HAM with glutamine and supplemented with 10% FCS. Human colonic carcinoma Caco-2 cells and human breast carcinoma MDA-MB-231 cells were grown in Dulbecco's modified eagle medium (DMEM) supplemented with 10% FCS. All cell lines grew as a monolayer, except for MAC16, which grew in suspension culture. For cell growth assays, cells were seeded either at 0.5 (MAC13, MAC26, A549, PC-3, DU-145 and MDA-MB-231) or $2.0 \times 10^4$ cells per well (MAC16 and Caco-2) and left for 2 h before drug addition. CV-6504 was dissolved in water. Cell numbers were determined 72 h after drug addition using a Coulter Counter model ZM.

### Fatty acid analysis

Total lipids were extracted from tumour and liver by the method of Folch et al (1957). Samples were homogenized in 10 volumes of chloroform:methanol (2:1v/v) containing an internal standard of margaric acid (50 μg) and butylated hydroxytoluene (0.01 volume dissolved in 50% ethanol). Organic and aqueous layers were separated by the addition of 0.2 volumes of distilled water and 1 ml of methanol, followed by centrifugation at 1500 *g* for 5 min. The organic layer was saponified by heating to 100°C in 2.5 ml of 5% sodium hydroxide in 50% methanol under nitrogen for 45–60 min. The samples were cooled and acidified to pH 2 with concentrated hydrochloric acid. The extracted fatty acids were then methylated by heating to 90°C for 5 min with 14% $BF_3$ in methanol. The cooled mixture was then extracted twice with 2 ml of hexane: chloroform (4:1, v/v). The fatty acid methyl esters were analysed using a Hewlett Packard 5890 Series II gas–liquid chromatograph connected to a Hewlett Packard HP3396A integrator. The gas chromatograph was fitted with a 30 m DB-23 narrow bore capillary column. The injector temperature was set at 190°C and the flame ionization detector at 240°C. The column head pressure was at 21 psi, with a split ratio of between 5 and 8 m $min^{-1}$. Samples were run on a temperature-programmed run with the initial temperature at 180°C for 5 min, followed by a 5°C per min rise in temperature to 220°C, which was held for 15 min. The peaks were identified by comparison of the retention times with those of authentic standards.

### Analysis of lipoxygenase metabolites of arachidonate

Cells ($5 \times 10^6$) were incubated with CV-6504 for 24 h before labelling in RPMI-1640 media containing normal serum levels. The cells were washed with phosphate-buffered saline (PBS) and resuspended in fresh medium containing 2.5 μCi of [$^3$H]arachidonic acid (specific activity 8.18 TBq $mmol^{-1}$; Du Pont, Herts UK) and mixed with unlabelled arachidonic acid to a final concentration of 10 μM. After 30 min (A549), 1h (Caco-2 and DU-145) or 2 h (MAC16, MAC13 and MAC26) at 37°C, the incubation was terminated by the addition of 1N hydrochloric acid to acidify the cell suspension to pH 3.5. These time intervals were chosen to allow maximum incorporation of [$^3$H]arachidonic acid into the cells. The cells were separated by low-speed centrifugation (1500 × *g*, 10 min) and were washed twice with PBS. The cells were resuspended in PBS (0.8 ml) and sonicated for 4 × 15 s on ice. The solution was acidified to pH 3.5 with 1N hydrochloric acid and chloroform: methanol (1:2, v/v) (3 ml) was added, followed by vigorous mixing for 1 min. After 30 min at room temperature, chloroform (1 ml) was added, and, after vigorous mixing, was followed by the addition of 0.001N hydrochloric acid (1 ml) and vortexing for another 10 s. After centrifugation at 2000 × *g* for 20 min at 4°C, the chloroform layer was removed and the aqueous phase was re-extracted with chloroform (2 ml). The combined chloroform extracts were evaporated under a stream of nitrogen and the residue was dissolved in acetonitrile (0.1 ml) and stored under argon at –70°C in the absence of light. Cell lipids were analysed by reversed-phase high-performance liquid chromatography (RP-HPLC) with a Waters μ Bondapak $C_{18}$ column (3.9 × 300 mm) by an isocratic elution at 1.5 ml $min^{-1}$ with 58% acetonitrile:water:acetic acid (20:100:0.05 v/v) and 42% acetonitrile – acetic acid. (100:0.05 v/v) (Liu et al, 1994). Radioactivity and ultraviolet absorbance at 237 nm were monitored Peaks were identified based on the retention times of authentic 5-, 12- and

Figure 1 Effect of daily administration of CV-6504 (10 mg $kg^{-1}$) (□) on tumour growth (A) and weight loss (B) in female NMRI mice ($n$ = 9 per group) bearing the MAC16 tumour. Control animals (X) received water alone. Both tumour volume and host body weight were normalized to 100% on day 1, the start of the experiment. A dose of 10 mg $kg^{-1}$ CV-6504 has previously been shown to be optimal against the MAC16 tumour in male mice (Hussey et al, 1996). Differences from control values are shown as a, $P < 0.05$ and b, $P < 0.01$

© Cancer Research Campaign 1997

**Figure 2** Effect of daily administration of CV-6504 (10 mg kg$^{-1}$) for 7 days on the fatty acid profile of control (□) and treated (▨) tumour (**A**) and liver (**B**). Differences from control values are shown as $^{a}P < 0.05$ and $^{b}P < 0.01$

**Table 1** Effect of CV-6504 on growth of cell lines in vitro

| Cell line | $IC_{50}$ (μM)[a] |
|---|---|
| MAC13 | 3 ± 1 |
| MAC16 | 3 ± 1 |
| MAC26 | 7 ± 1 |
| A549 | 61 ± 1 |
| Caco-2 | 5 ± 0 |
| MDA-MB-231 | 10 ± 1 |
| PC-3 | 17 ± 0 |
| DU-145 | 70 ± 0 |

[a]Concentration producing 50% inhibition of cell growth over a 72-h period. The values are averages of nine determinations.

**Figure 3** The effect of 10 μM CV-6504 on the metabolism of AA to 5-, 12- and 15-HETE in (**A**) MAC13 (□), MAC16 (▧), MAC26 (■) and (**B**) A549 (□), Caco-2 (▧) and DU-145 (■). The top box for each cell line represents the control and the bottom box the treated cells. The experiment was repeated four times. Differences from controls are shown as $^{a}P < 0.05$ and $^{b}P < 0.01$

15-HETE (Sigma Chemical Co., Poole, Dorset, UK). The amounts of HETEs were quantified based on the specific activity of radiolabelled arachidonic acid and the ratio of radiolabelled to unlabelled substrate.

### Statistical analysis

Results are presented as means ± s.e.m. The data were statistically evaluated using two-way analysis of variance followed by Tukey's test.

### RESULTS

Growth of the MAC16 tumour in NMRI mice is accompanied by a progressive cachexia, which increases as tumour growth increases (Beck and Tisdale, 1987). The effect of daily oral administration of CV-6504 (10 mg kg$^{-1}$) on tumour growth and cachexia in this murine model in female NMRI mice is shown in Figure 1A and B. Although the tumour was already established when therapy was initiated, CV-6504 produced a reduction in tumour volume from day 11 (Figure 1A) and this was accompanied by a significant reduction in host body weight loss, which was significant from day 13 (Figure 1B). Since the control animals had to be terminated owing to cachexia on day 15, CV-6504 effectively prolonged the survival of mice bearing this tumour.

Since CV-6504 may be expected to interfere with lipid metabolism, the effect of this agent administered daily for 7 days at 10 mg $kg^{-1}$ on the fatty acid composition of the liver and the tumour was investigated (Figure 2A and B). CV-6504 reduced the total fatty acid content of the liver with specific reductions in AA, LA and oleic acid (OA) (Figure 2A). The total fatty acid content of the tumour was also significantly reduced with specific reductions in AA, LA, OA, stearic and palmitic acids (Figure 2B). These results confirm that CV-6504 modulates fatty acid metabolism in vivo.

Further mechanistic studies were performed on cells in vitro. The effect of CV-6504 on the growth of murine and human colon, breast, lung and prostatic tumours is shown in Table 1. The sensitivity varied between the cell lines with human colon (Caco-2), breast (MDA-MB-231) and prostate (PC-3) being of the same order as for the MAC cell lines. In contrast, the human lung (A549) and prostate (DU-145) were more resistant with $IC_{50}$ values for CV-6504 being of the order of tenfold greater.

In order to investigate mechanisms of sensitivity and resistance, the effect of CV-6504 on the metabolism of AA has been investigated in the three human cell lines, A549, DU-145 and Caco-2, and compared with that in the MAC cell lines, MAC16, MAC13 and MAC26. All six cell lines showed detectable formation of 5-, 12- and 15-HETE from a pulse of $[^3H]$AA (Figure 3). However, the level of incorporation of $[^3H]$AA was low for MAC16, and both DU-145 and A549 also showed a low level of formation of 5-, 12- and 15-HETE. For most cell lines, the distribution of AA between the 5-, 12- and 15-lipoxygenase pathways was approximately the same, although 5-HETE formation exceeded the 12- and 15-HETE in MAC13 cells. The effect of preincubation with 10 μM CV-6504 on the metabolism of AA through the lipoxygenase pathways is also shown in Figure 3. In cell lines sensitive to growth inhibition by CV-6504, MAC16, MAC13, MAC26 and Caco-2, there was a significant reduction in 5-, 12- and 15-HETE production, and for MAC16, MAC13 and Caco-2, a significant reduction in unmetabolized AA recovered relative to untreated cells. At a concentration of 10 μM, CV-6504 had no effect on either 5-, 12- or 15-HETE production in either A549 or DU-145 cells.

### DISCUSSION

The sensitivity of the various cell lines to growth inhibition by CV-6504 appears to correlate with the requirement of LA for growth. Thus, MAC cell lines have been shown to undergo growth stimulation by LA both in vitro and in vivo (Hussey and Tisdale, 1994) and these cell lines show low $IC_{50}$ values for CV-6504. The oestrogen-independent human breast cancer cell line, MDA-MB-231, is also sensitive to CV-6504. LA has been shown to stimulate proliferation of this cell line (Rose and Connolly, 1989), and this was dependent on the products of lipoxygenase rather than cyclooxygenase pathways (Rose and Connolly, 1990). When this cell line was transplanted into nude mice, diets rich in *n*-3 PUFAs reduced both tumour growth and metastasis, and this was found to correlate with a three- to four-fold reduction in 12- and 15-HETE production (Rose et al, 1995). Growth of the androgen-unresponsive prostatic cancer cell line, PC-3, was shown to be stimulated in vitro by LA, while DU-145, which is also androgen unresponsive, showed no growth response to LA (Rose and Connolly, 1991). In this study, we have shown CV-6504 to have $IC_{50}$ values of 17 and 70 μM against the cell lines PC-3 and DU-145 respectively.

The effect of CV-6504 on PUFA metabolism has been examined by determining the effect on conversion of AA to 5-, 12- and 15-HETE in MAC16, MAC13, MAC26, Caco-2, DU-145 and A549 cell lines. Both A549 and DU-145 were relatively resistant to growth inhibition by CV-6504 in comparison with the other cell lines. At a concentration of 10 μM, CV-6504 was capable of causing significant inhibition of 5-, 12- and 15-HETE production in MAC16, MAC13, MAC26 and Caco-2 cells, but in both A549 and DU-145, metabolism of AA through the 5-, 12- and 15-lipoxygenase pathways was not affected by this concentration of CV-6504. Thus, the $IC_{50}$ values for CV-6504 against these five cell lines correlate with the inhibition of AA metabolism through the three lipoxygenase pathways.

Although CV-6504 was designed as a specific 5-lipoxygenase inhibitor (Ohkawa et al, 1991), these results show that it is also capable of profound inhibition of both the 12- and 15-lipoxygenase pathways in sensitive tumour cell lines. Another agent inhibiting tumour proliferation, eicosapentaenoic acid (EPA) has been shown to reduce the tumour concentration of 12- and 15-HETE, while the level of 5-HETE was unaffected (Rose et al, 1995). These results suggest that inhibition of 12- and/or 15-lipoxygenases may be most important for tumour growth inhibition, and some 5-lipoxygenase inhibitors may show anti-tumour activity as a result of cross-reactivity towards these pathways.

12-HETE has been shown to stimulate DNA synthesis in fetal bovine aortic endothelial cells (Setty et al, 1987), which is possibly mediated through diacylglycerol kinase inhibition and the concomitant accumulation of cellular diacylglycerol. This may explain the ability of 12-HETE to activate protein kinase C in tumour cells by stimulating translocation of the enzyme to the cell membrane (Honn et al, 1994). 12-HETE may also mediate EGF/insulin-stimulated DNA synthesis in neonatal rat lens epithelial cells by regulating proto-oncogene expression (Lysz et al, 1994). Friend erythroleukaemic cells in logarithmic phase metabolize higher concentrations of 15-HETE compared with cells in stationary phase (Postaok et al, 1990). If 15-HETE production is inhibited, DNA synthesis is also inhibited, suggesting a role for 15-HETE in the proliferation of these cells.

Thus, some, but not all, tumours appear to depend on these metabolites of AA for their continual proliferation. Such tumours may be expected to display sensitivity towards CV-6504. Even more effective anti-tumour agents could be generated by specific inhibitors of the 12- and/or 15-lipoxygenase pathways.

### ACKNOWLEDGEMENTS

We thank Mr M Wynter for the tumour transplantations. This work was supported by Takeda Chemical Industries Ltd, Osaka, Japan.

### REFERENCES

Bandyopadhyay GK, Imagawa W, Wallace DR and Nandi S (1988) Proliferative effects of insulin and epidermal growth factor on mouse mammary epithelial cells in primary culture. Enhancement by hydroxyeicosatetraenoic acids and synergism with prostaglandin $E_2$. *J Biol Chem* **263**: 7567–7573

© Cancer Research Campaign 1997

Beck SA and Tisdale MJ (1987) Production of lipolytic and proteolytic factors by a murine tumor-producing cachexia in the host. *Cancer Res* **47**: 5919–5923

Bibby MC, Double JA, Ali SA, Fearon KCH, Brennan RA and Tisdale MJ (1987) Characterisation of a cachectic transplantable adenocarcinoma of the mouse colon. *J Natl Cancer Inst* **78**: 539–546

Buckman DK, Hubbard NE and Erickson KL (1991) Eicosanoids and linoleate-enhanced growth of mouse mammary tumor cells. *Prostaglandins Leukotrienes Ess Fatty Acids* **44**: 177–184

Eling TE and Glasgow WC (1994) Cellular proliferation and lipid metabolism: importance of lipoxygenases in modulating epidermal growth factor-dependent mitogenesis. *Cancer Metast Rev* **13**: 397–410

Folch J, Less M and Stanley GHS (1957) A simple method for the isolation and purification of total lipids from animal tissues. *J Biol Chem* **226**: 497–509

Honn KV, Tang DG, Gao X, Butovich IA, Liu B, Timar J and Hagmann W (1994) 12-Lipoxygenases and 12(S)-HETE: role in cancer metastasis. *Cancer Metast Rev* **13**: 365–396

Hussey HJ and Tisdale MJ (1994) Effect of polyunsaturated fatty acids on the growth of murine colon adenocarcinomas *in vitro* and *in vivo*. *Br J Cancer* **70**: 6–10

Hussey HJ, Bibby MC and Tisdale MJ (1996) Novel antitumour activity of 2, 3, 5-trimethyl-6-(3-pyridylmethyl)-1,4-benzoquinone (CV-6504) against established murine adenocarcinomas (MAC). *Br J Cancer* **73**: 1187–1192

Liu B, Marnett LJ, Chaudhary A, Ji C, Blair IA, Johnson CR, Diglio CA and Honn KV (1994) Biosynthesis of 12(S) hydroxyeicosatetraenoic acid by B16 amelanotic melanoma cells is a determinant of their metastatic potential. *Lab Invest* **70**: 314–323

Lysz TW, Arora JK, Lin C and Zelenka PS (1994) 12(S)-Hydroxyeicosatetraenoic acid regulates DNA synthesis and protooncogene expression induced by epidermal growth factor and insulin in rat lens epithelium. *Cell Growth Different* **5**: 1069–1076

Ohkawa S, Terao S, Terashita Z, Shibouta Y and Nishikana K (1991) Dual inhibitors of thromboxane $A_2$ synthase and 5-lipoxygenase with scavenging activity of active oxygen species. Synthesis of a novel series of (3-pyridylmethyl) benzoquinone derivatives. *J Med Chem* **34**: 267–276

Postaok O, Nystuen L, King L, Veno M and Beckman BS (1990) 15-Lipoxygenase products of arachidonate play a role in the proliferation of transformed erythroid cells. *Am J Physiol* **259**: C849–C853

Reddy BS and Masura Y (1984) Tumor promotion by dietary fat in azoxymethane-induced colon carcinogenesis in female F344 rats: influence of amount and sources of dietary fat. *J Natl Cancer Inst* **72**: 745–750

Rose DP and Connolly JM (1989) Stimulation of growth of human breast cancer cell lines in culture by linoleic acid. *Biochem Biophys Res Commun* **64**: 277–283

Rose DP and Connolly JM (1990) Effects of fatty acids and inhibitors of eicosanoid synthesis on the growth of a human breast cancer cell line in culture. *Cancer Res* **50**: 7139–7144

Rose DP and Connolly JM (1991) Effects of fatty acids and eicosanoid synthesis inhibitors on the growth of two human prostate cancer cell lines. *Prostate* **18**: 243–254

Rose DP, Connolly JM and Meschter CL (1991) Effects of dietary fat on human breast cancer growth and lung metastasis in nude mice. *J Natl Cancer Inst* **83**: 1491–1495

Rose DP, Connolly JM, Rayburn J and Coleman M (1995) Influence of diets containing eicosapentaenoic or docosahexaenoic acid on growth and metastasis of breast cancer cells in nude mice. *J Natl Cancer Inst* **87**: 587–592

Setty BNY, Graeber JE and Stuart MJ (1987) The mitogenic effect of 15-and 12-hydroxyeicosatetraenoic acid on endothelial cells may be mediated via diacylglycerol kinase inhibition. *J Biol Chem* **262**: 17613–17622

Shibouta Y, Terashita Z-I, Imura Y, Shino A, Kawamura M, Ohtsuki K, Ohkawa S, Nishikawa K and Fujiwara Y (1991) Involvement of thromboxane $A_2$ leukotrienes and free radicals in puromycin nephrosis in rats. *Kidney Int* **39**: 920–929

Wicha MS, Liotta LA and Kidwell WR (1979) Effects of free fatty acids on the growth of normal and neoplastic rat mammary epithelial cells. *Cancer Res* **39**: 426–435

© Cancer Research Campaign 1997